# Immunologic targeting of CD30 eliminates tumourigenic human pluripotent stem cells, allowing safer clinical application of hiPSC-based cell therapy

**DOI:** 10.1038/s41598-018-21923-8

**Published:** 2018-02-27

**Authors:** Nagako Sougawa, Shigeru Miyagawa, Satsuki Fukushima, Ai Kawamura, Junya Yokoyama, Emiko Ito, Akima Harada, Kaori Okimoto, Noriko Mochizuki-Oda, Atsuhiro Saito, Yoshiki Sawa

**Affiliations:** Department of Cardiovascular Surgery, Osaka University Graduate School of Medicine, Suita, Osaka, Japan

## Abstract

Induced pluripotent stem cells (iPSCs) are promising candidate cells for cardiomyogenesis in the failing heart. However, teratoma/tumour formation originating from undifferentiated iPSCs contaminating the graft is a critical concern for clinical application. Here, we hypothesized that brentuximab vedotin, which targets CD30, induces apoptosis in tumourigenic cells, thus increasing the safety of iPSC therapy for heart failure. Flow cytometry analysis identified consistent expression of CD30 in undifferentiated human iPSCs. Addition of brentuximab vedotin *in vitro* for 72 h efficiently induced cell death in human iPSCs, associated with a significant increase in G2/M phase cells. Brentuximab vedotin significantly reduced *Lin28* expression in cardiomyogenically differentiated human iPSCs. Transplantation of human iPSC-derived cardiomyocytes (CMs) without treatment into NOG mice consistently induced teratoma/tumour formation, with a substantial number of Ki-67–positive cells in the graft at 4 months post-transplant, whereas iPSC-derived CMs treated with brentuximab vedotin prior to the transplantation did not show teratoma/tumour formation, which was associated with absence of Ki-67–positive cells in the graft over the same period. These findings suggest that *in vitro* treatment with brentuximab vedotin, targeting the CD30-positive iPSC fraction, reduced tumourigenicity in human iPSC-derived CMs, potentially providing enhanced safety for iPSC-based cardiomyogenesis therapy in clinical scenarios.

## Introduction

Cell-based therapy is one of the options for treating heart failure, which is the leading cause of morbidity and mortality worldwide. Human induced pluripotent stem cells (hiPSCs), which have the ability to differentiate into several cell types^1^, are promising cell sources and have already exhibited efficacy in experimental models^2–5^. However, the main drawback in hiPSC therapy is the risk of tumour formation caused by immature cells contaminating the grafts^6–8^, suggesting that the success of hiPSC-based cell therapy is dependent on controlling tumourigenicity after implantation. Several strategies to remove residual undifferentiated hiPSCs from differentiated cell cultures, including transfection of suicide genes into hiPSCs^9^, use of chemical inhibitors^10–13^, cell sorting using hiPSC-specific antibodies^14,15^, and glucose deprivation in the cell culture medium^16^, have been reported. Although cell sorting and glucose deprivation strategies may be feasible, they can also reduce cell viability and numbers. Therefore, alternative strategies to prevent tumour formation should be considered for clinical application.

Recently, antibody-based therapies directed against unique antigens expressed on cancer cells have been successfully developed and have shown significant therapeutic effects in the clinical treatment of cancer^17^. Therefore, we propose that antibody-based therapies may also be able to eliminate immature hiPSCs.

In this study, we address the following specific questions. (1) Do hiPSCs have a specific surface marker that is not expressed by differentiated cardiomyocytes? (2) Can an antibody-cytotoxic drug conjugate targeting the specific marker eliminate residual undifferentiated cells from hiPSC derivatives that were cardiomyogenically differentiated? (3) Can the antibody-cytotoxic drug conjugate provide complete control of tumourigenicity *in vivo*, resulting in enhanced safety in the clinical application of hiPSC-based cell therapy for heart disease? The present study demonstrates that brentuximab vedotin, which targets a specific antigen on hiPSCs, is an efficient and specific hiPSC-eliminating agent, suggesting its promising application in the near future for clinical use in cell therapy.

## Results

### CD30 was selectively expressed in hiPSCs

**Figure 1:**
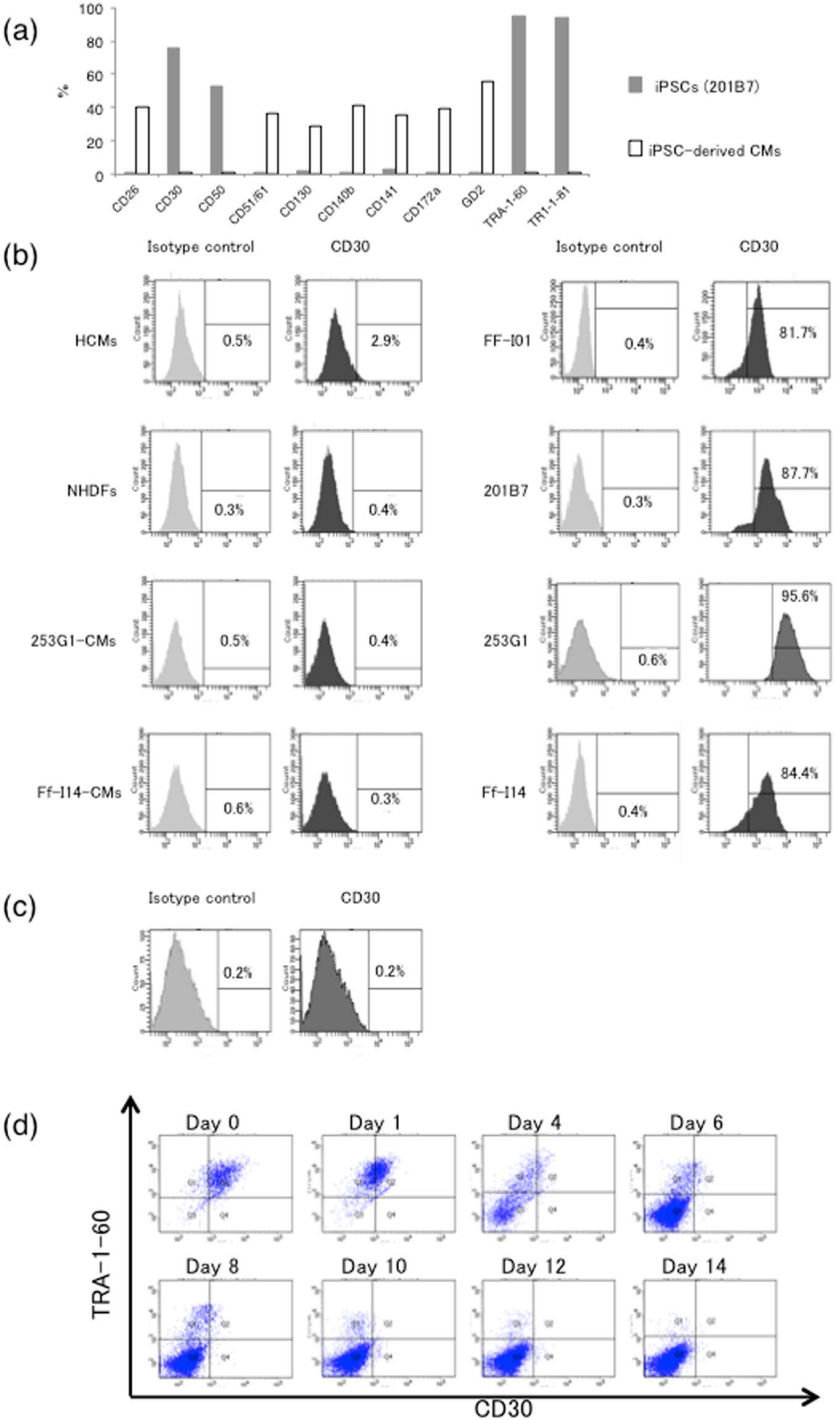
Expression of CD30 on hiPSCs (**a**) Expression of surface markers was compared between hiPSCs and hiPSC derived-CMs. The data are representative of at least three independent experiments. (**b**) Expression of CD30 on several cell surfaces. The number in the histograms indicates the percentage of CD30-positive cells. The data are representative of at least three independent experiments. (**c**) Expression of CD30 on hiPSC-derived CMs. The number in the histograms indicates the percentage of CD30-positive cells. The data are representative of at least three independent experiments. (**d**) Down-regulation of CD30 expression during hiPSC differentiation into CMs. The data are representative of at least three independent experiments.

To identify the unique cell surface marker on hiPSCs, we first compared surface marker expression patterns between hiPSCs and hiPSC-derived CMs. The expression profile showed that CD30, TRA-1-60, and TRA-1-81 were highly expressed on hiPSCs but not on hiPSC-derived CMs (hiPSCs vs. hiPSC-derived CMs, CD30; 91.2 ± 10.9% vs. 0.4 ± 0.4% *p* < 0.001, TRA-1-60; 98.3 ± 2.3% vs. 3.7 ± 4.0% *p* < 0.05, TRA-1-81; 98.3 ± 2.3% vs. 3.0 ± 3.1%, *p* < 0.01) (Fig. 1a). In addition, FACS analysis revealed that CD30 was highly expressed on hiPSC clones (81.7–95.2%), whereas CD30 was not detected on differentiated cells, including human cardiomyocytes (HCMs), normal human dermal fibroblasts (NHDFs), and hiPSC-derived CMs (253G1-CMs, Ff-I14-CMs) (Fig. 1b). Furthermore, 253G1-derived CMs yielded by another methods^18^ also showed no expression of CD30 (Fig. 1c). To investigate how rapidly CD30 is down-regulated during the differentiation of hiPSCs, we examined its expression during the differentiation process. At Day 4 of induction of differentiation, 29.3 ± 3.3% of the cells were TRA-1-60 positive, whereas only 6.1 ± 1.1% of the cells were CD30 positive (*p* < 0.001). In contrast, at Day 16, the rate of TRA-1-60–positive cells was approximately equal to that of CD30-positive cells (Fig. 1d). This analysis indicated that CD30 was markedly down-regulated during the first week of differentiation, and the down-regulation of CD30 was faster than that of TRA-1-60.

### Brentuximab vedotin induced cell death in hiPSCs

**Figure 2:**
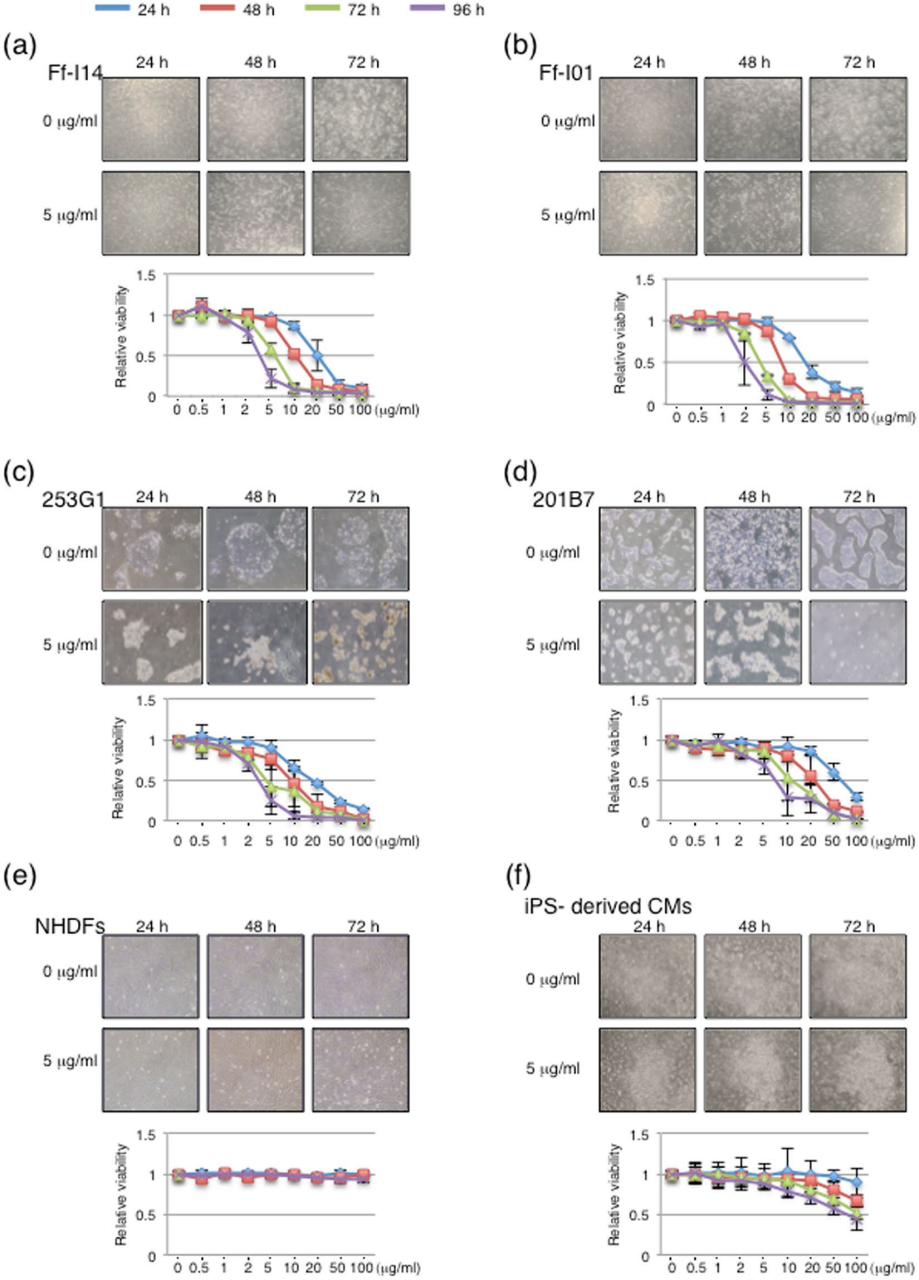
Effect of brentuximab vedotin on viability of hiPSCs and differentiated cells (**a**) Ff-I14 cells, (**b**) Ff-I01 cells, (**c**) 253G1 cells, (**d**) 201B7 cells, (**e**) NHDFs, and (**f**) iPSC-derived CMs were treated with brentuximab vedotin at 0, or 5 μg/ml for 24, 48, and 72 h. Panels are representative phase-contrast images of cells. The relative viabilities of cells in response to brentuximab vedotin treatment were determined using CCK-8 solution. Graph data were repeated at least three times independently.

Having demonstrated that CD30 was selectively expressed on hiPSCs (Fig. 1), we used the antibody-drug conjugate brentuximab vedotin (Adcetris^TM^), which targets CD30-positive cells. Brentuximab vedotin induced < 50% cell death of undifferentiated Ff-I14 cells at 20 μg/ml when applied for 24 h, 10 μg/ml for 48 h, and 5 μg/ml for 72 h (*p* < 0.001), and > 85% cell death of Ff-I14 cells at 50 μg/ml when applied for 24 h, 20 μg/ml for 48 h, 10 μg/ml for 72 h, and 5 μg/ml for 96 h (*p* < 0.001) (Fig. 2a). Similarly, brentuximab vedotin induced robust cell death of undifferentiated Ff-I01 (Fig. 2b), 253G1 (Fig. 2c), and 201B7 (Fig. 2d) cells. In contrast, brentuximab vedotin did not induce any cell death in NHDFs, even at 100 μg/ml for 96 h. (*p* > 0.05) (Fig. 2e). In the case of hiPSC-derived CMs treated with brentuximab vedotin, there was apparently no significant difference in the hiPSC-derived CMs with or without brentuximab vedotin. The relative viability of cells slightly decreased when treated with brentuximab vedotin at 10 μg/ml for 72 h compared to untreated cells. Brentuximab vedotin applied at 20 μg/ml for 72 h significantly induced cell death in hiPSC-derived CMs (Fig. 2f).

### Brentuximab vedotin induced G2/M arrest in hiPSCs

**Figure 3:**
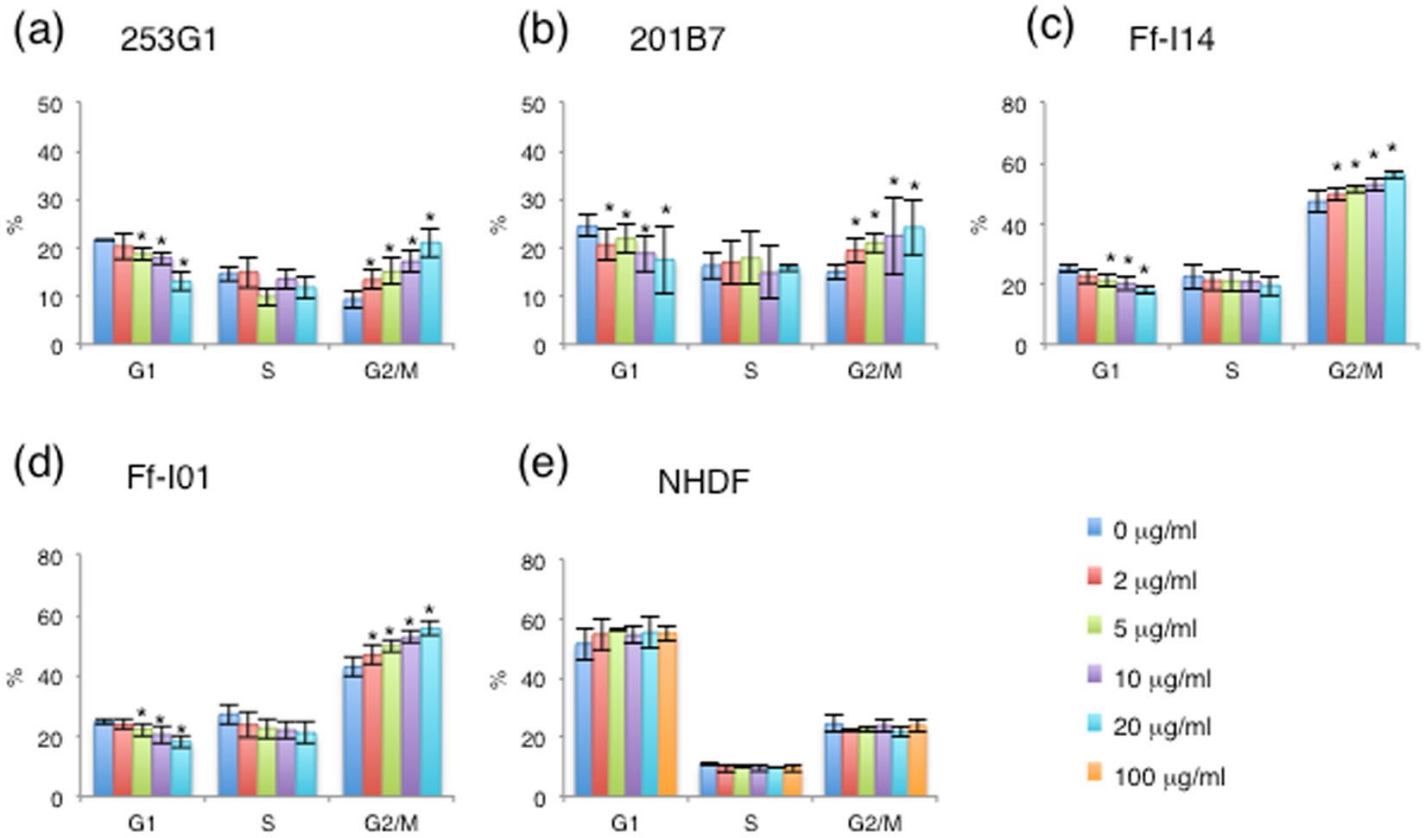
Effect of brentuximab vedotin on cell cycle (**a**) 253G1 cells, (**b**) 201B7 cells, (**c**) Ff-I14 cells, (**d**) Ff-I01 cells, and (**e**) NHDFs were treated with increasing concentrations of brentuximab vedotin. At 10 h after treatment, cells were stained with propidium iodide (PI) to detect DNA content and analysed by flow cytometry. Bar graphs indicate the percentage of cells in different phases of the cell cycle. Data were collected from at least three independent experiments. *p < 0.05 vs. 0 μg/ml.

Given that brentuximab vedotin inhibits microtubule polymerization during the G2/M phase of the cell cycle^19^, we evaluated whether this phenomenon occurred in hiPSCs. In contrast to the untreated control, 253G1 cells exposed to 2, 5, 10, or 20 μg/ml brentuximab vedotin showed an increase in the fraction of cells in the G2/M phase (from 9.3 ± 1.1% to 13.3 ± 2.0%, 15.0 ± 2.7%, 17.1 ± 2.3%, or 21.1 ± 3.0%, respectively) with a concomitant decrease in the fraction of G1 cells (from 21.6 ± 1.5% to 20.3 ± 2.6%, 18.7 ± 1.4%, 17.9 ± 1.3%, or 13.1 ± 1.9%, respectively) (Fig. 3a). Similar results were observed in the following cells exposed to 2, 5, 10, or 20 μg/ml brentuximab vedotin: 201B7 cells (G2/M phase; from 15.0 ± 1.6% to 19.4 ± 2.7%, 21.0 ± 1.9%, 22.5 ± 7.9%, or 24.2 ± 5.7%, respectively, G1 phase; from 24.5 ± 2.2% to 20.6 ± 3.1%, 21.9 ± 3.0%, 18.8 ± 3.8%, or 17.5 ± 7.0%, respectively) (Fig. 3b), Ff-I14 cells (G2/M phase; from 47.3 ± 3.6% to 49.7 ± 1.9%, 51.4 ± 1.1%, 52.3 ± 2.1%, or 56.2 ± 1.2%, respectively, G1 phase; from 25.0 ± 1.0% to 22.6 ± 2.3%, 21.0 ± 2.0%, 20.1 ± 2.1%, or 17.9 ± 1.3%, respectively) (Fig. 3c), and Ff-I01 cells (G2/M phase; from 42.9 ± 3.2% to 46.7 ± 3.3%, 49.8 ± 1.9%, 52.6 ± 1.9%, or 55.6 ± 2.1%, respectively, G1 phase; from 24.6 ± 0.9% to 23.8 ± 1.7%, 22.2 ± 2.2%, 20.3 ± 2.8%, or 18.1 ± 1.9%, respectively) (Fig. 3d). Thus hiPSCs demonstrated a concentration-dependent increase in the fraction of cells in the G2/M phase in response to brentuximab vedotin treatment. However, in contrast to untreated NHDFs, NHDFs exposed to 20 or 100 μg/ml brentuximab vedotin showed no increase in the fraction of G2/M cells (24.0 ± 2.9% vs. 21.8 ± 1.8% or 24.1 ± 2.3%, respectively) (*p* > 0.1); thus, this effect was not observed with NHDFs at any concentration (Fig. 3e).

### Brentuximab vedotin induced apoptosis of hiPSCs

**Figure 4:**
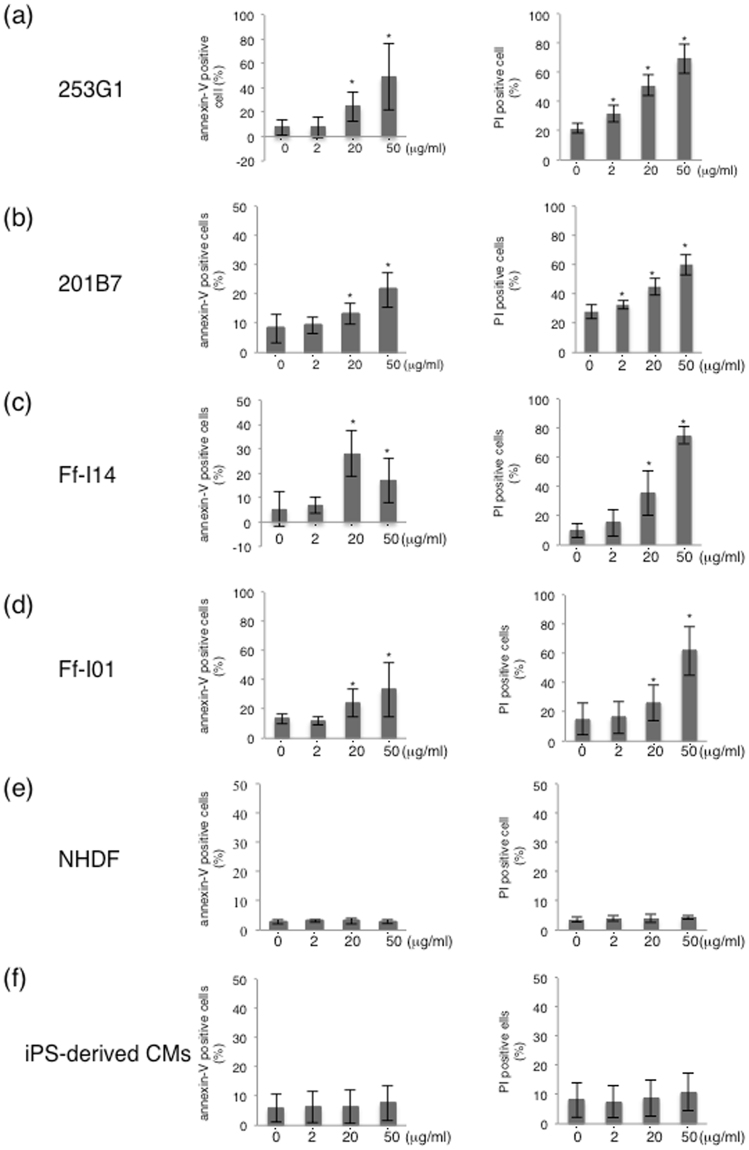
Effect of brentuximab vedotin on apoptosis of hiPSCs (**a**) 253G1 cells, (**b**) 201B7 cells, (**c**) Ff-I14 cells, (**d**) Ff-I01 cells, (**e**) NHDFs, and (**f**) iPSC-derived CMs were treated with increasing concentrations of brentuximab vedotin. At 48 h after treatment, apoptotic cells were determined by flow cytometry following annexin-V staining and PI staining. Bar graphs indicate the percentage of annexin-V–positive cells and PI-positive cells. Data were collected from at least three independent experiments. *p < 0.05 vs. 0 μg/ml.

We next examined the effect of brentuximab vedotin on cellular apoptosis. Significantly increased apoptosis was observed in the tested hiPSC lines treated at 20 μg/ml and 50 μg/ml for 48 h (Fig. 4) compared to untreated hiPSCs. 253G1 cells showed 24.7 ± 12.2% (*p* < 0.05) annexin-V–positive cells and 51.3 ± 7.5% (*p* < 0.05) PI-positive cells. In addition, exposure to 50 μg/ml brentuximab vedotin resulted in 49.0 ± 27.0% (*p* < 0.05) annexin-V–positive cells and 69.4 ± 10.4% (*p* < 0.05) PI-positive cells (Fig. 4a). Similar results were observed in 201B7, Ff-I14, and Ff-I01 cells. 201B7 cells exposed to 20 or 50 μg/ml brentuximab vedotin showed 13.2 ± 3.5% (*p* < 0.05) and 21.4 ± 5.9% (*p* < 0.05) annexin-V–positive cells, respectively, and showed 45.1 ± 5.7% (*p* < 0.05) and 60.3 ± 7.1% (*p* < 0.05) PI-positive cells, respectively (Fig. 4b). Ff-I14 cells exposed to 20 or 50 μg/ml brentuximab vedotin showed 28.0 ± 9.5% (*p* < 0.05) and 17.1 ± 9.4% (*p* < 0.05) annexin-V–positive cells, respectively, and 35.5 ± 15.0% (*p* < 0.05) and 75.2 ± 5.7% (*p* < 0.05) PI–positive cells, respectively (Fig. 4c). Ff-I01 cells exposed to 20 or 50 μg/ml brentuximab vedotin showed 13.4 ± 3.4% (*p* < 0.05) and 33.3 ± 18.9% (*p* < 0.05) annexin-V–positive cells, respectively, and showed 26.4 ± 12.3% (*p* < 0.05) and 61.9 ± 16.7% (*p* < 0.05) PI–positive cells, respectively (Fig. 4d). In contrast, NHDF cells showed no increase of annexin-V–positive cells and PI-positive cells at any concentration of brentuximab vedotin (Fig. 4e). In the case of hiPSC-derived CMs treated with brentuximab vedotin, the percentage of annexin-V-positive cells and PI-positive cells tended to increase concentration-dependently (Annexin-V- positive cells; 0 μg/ml; 5.8 ± 4.8%, 2 μg/ml; 6.3 ± 5.3%, 20 μg/ml; 6.3 ± 5.8% and 50 μg/ml; 7.7 ± 6.1%, PI-positive cells; 0 μg/ml; 8.1 ± 5.9% and 2 μg/ml; 7.5 ± 5.5%, 20 μg/ml; 8.8 ± 6.3% and 50 μg/ml; 10.8 ± 6.5%). However, the difference was not significant (Fig. 4f).

### Brentuximab vedotin reduced the number of undifferentiated cells among hiPSC-derived CMs

**Figure 5:**
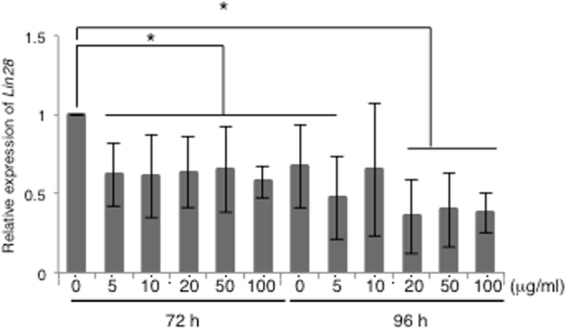
Effect of brentuximab vedotin on the expression of *Lin28.* Expression of *Lin28* in hiPSC-derived CMs after brentuximab vedotin treatment was determined by qRT-PCR analysis. hiPSC-derived CMs were treated with brentuximab vedotin at the indicated doses for 72 and 96 h. Total RNA was isolated from the cells. Y-axis indicates relative gene expression compared with non-treated hiPSC-derived CMs for 72 h. Data were collected from at least three independent experiments. *p < 0.05 vs. 72 h, 0 μg/ml.

We examined the effect of brentuximab vedotin on hiPSC-derived CMs. After we treated hiPSC-derived CMs with brentuximab vedotin for 72 or 96 h, we evaluated the fraction of residual undifferentiated cells by qRT-PCR. The amount of *Lin28*, which is a pluripotency marker, was decreased by 38.3 ± 20% in hiPSC-derived CMs treated with brentuximab vedotin at 5 μg/ml for 72 h, compared to untreated cells (*p* < 0.05). In addition, brentuximab vedotin at 5, 10, 20, 50, and 100 μg/ml reduced the level of *Lin28* by 39 ± 26.3%, 36 ± 22.5%, 48 ± 12.5%, and 46.3 ± 10.3%, respectively (*p* < 0.05). However, brentuximab vedotin concentrations of 20 μg/ml or higher did not significantly reduce *Lin28* expression when compared to 10 μg/ml brentuximab vedotin treatment (*p* > 0.1). The hiPSC-derived CMs treated with brentuximab vedotin at 10 μg/ml for 72 h showed a significant reduction of *Lin28* expression *in vitro*, in contrast to untreated hiPSC-derived CMs. However, higher concentrations and longer exposure of this drug did not induce further reduction of *Lin28* expression (reduction of *Lin28* expression with brentuximab vedotin treatment for 96 h: 5 μg/ml, 52.9 ± 26.3%; 10 μg/ml, 34.9 ± 41.9%; 20 μg/ml, 64.6 ± 23.3%; 50 μg/ml, 60.5 ± 23.3%; and 100 μg/ml, 62.3 ± 12.7%) (*p* > 0.05) (Fig. 5).

### Effect of brentuximab vedotin on cytotoxicity in hiPSC-derived CMs

**Figure 6:**
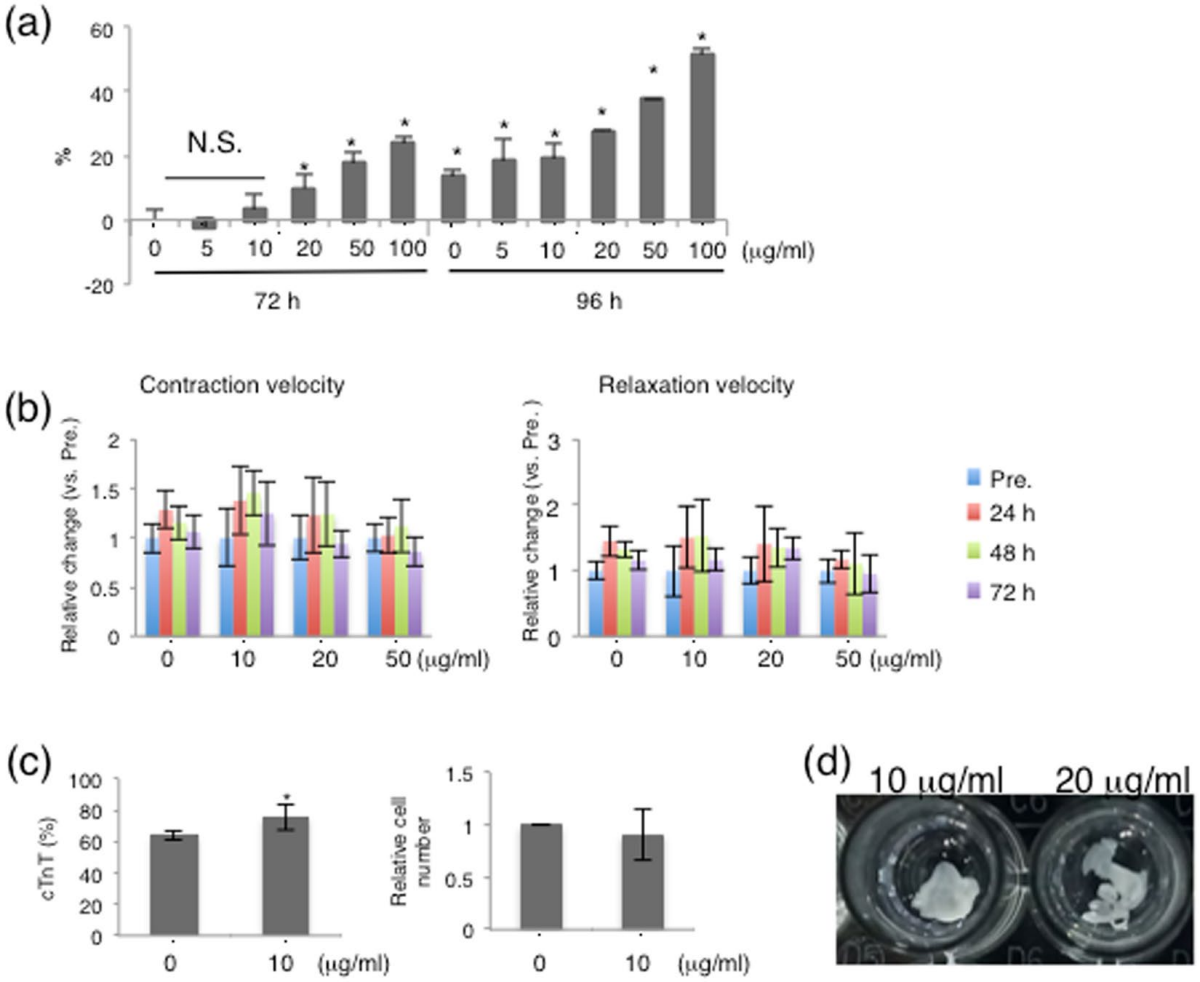
Cytotoxicity of brentuximab vedotin to hiPSC-derived CMs (**a**) Cytotoxicity of brentuximab vedotin in hiPSC-derived CMs was assessed by LDH assay. Y-axis indicates relative cytotoxicity compared with non-treated hiPSC-derived CMs for 72 h. Experiments were repeated at least thrice independently. *p < 0.01 vs. 72 h, 0 μg/ml. (**b**) Contraction and relaxation velocity of hiPSC-derived CMs was assessed by motion analyser system. Y-axis indicates relative change compared to pre-treatment hiPSC-derived CMs. (**c**) The percentage cTnT-expressing hiPSC-derived CMs and the relative number of hiPSC-derived CMs were assessed after they were cultured in normal medium for a week. *p < 0.05 vs. 72 h, 0 μg/ml. (**d**) Effect of brentuximab vedotin on cell sheet formation. At 72 h after starting treatment of hiPSC-derived CMs with brentuximab vedotin in a temperature-responsive plate, monolayered cell sheets were fabricated using cells treated with brentuximab vedotin at 10 μg/ml (left) and 20 μg/ml (right). Panels are representative macroscopic images of monolayered cell sheets.

Because brentuximab vedotin is an anticancer agent, we examined its side effects on hiPSC-derived CMs. Lactate dehydrogenase (LDH) release from hiPSC-derived CMs treated with brentuximab vedotin was increased in a concentration- and time-dependent manner. Brentuximab vedotin induced 3% or less LDH release at 5 or 10 μg/ml for 72 h compared to untreated cells (*p* > 0.05). However, treatment with over 20 μg/ml brentuximab vedotin for 72 h significantly induced LDH release (20 μg/ml, 9.7 ± 4.4%; 50 μg/ml, 17.3 ± 3.7%; and 100 μg/ml, 23.6 ± 2.6%) (*p* < 0.05). Moreover, treatment with brentuximab vedotin for 96 h showed remarkably higher LDH release than treatment for 72 h (5, 10, 20, 50, and 100 μg/ml; 18.2 ± 6.9%, 18.8 ± 5.0%, 27.4 ± 0.8%, 37.4 ± 0.5%, and 51.4 ± 2.0%, respectively) (Fig. 6a). Subsequently, to examine the effect of brentuximab vedotin on the function of hiPSC-derived CMs, we checked their contraction and relaxation velocity after treatment. *In vitro*, the contraction and relaxation velocity of hiPSC-derived CMs treated with brentuximab vedotin at 20 μg/ml was not significantly different from that of untreated hiPSC-derived CMs (Fig. 6b). Furthermore, we assessed the cytotoxicity over time. After treatment with brentuximab vedotin at 10 μg/ml for 72 h, we added one more week of culture in normal culture medium. hiPSC-derived CMs treated with brentuximab vedotin at 10 μg/ml showed 75.3 ± 7.9% cTnT-positive cells. In contrast, untreated hiPSC-derived CMs after one more week of culture in normal culture media showed 64.0 ± 3.3% cTnT-positive cells. In addition, the relative number of treated cells after the week of additional culture was 90.6 ± 0.2% of that of untreated cells. However, the difference was not significant (Fig. 6c). We evaluated sheet formation after brentuximab vedotin treatment. When hiPSC-derived CMs were treated with brentuximab vedotin at 10 μg/ml for 72 h, cell sheets were obtained. However, treatment with 20 μg/ml brentuximab vedotin for 72 h occasionally weakened cell–cell contact and cell sheets were not obtained (Fig. 6d).

### Brentuximab vedotin prevented teratoma/tumour formation after transplantation of hiPSC-derived CMs

**Figure 7:**
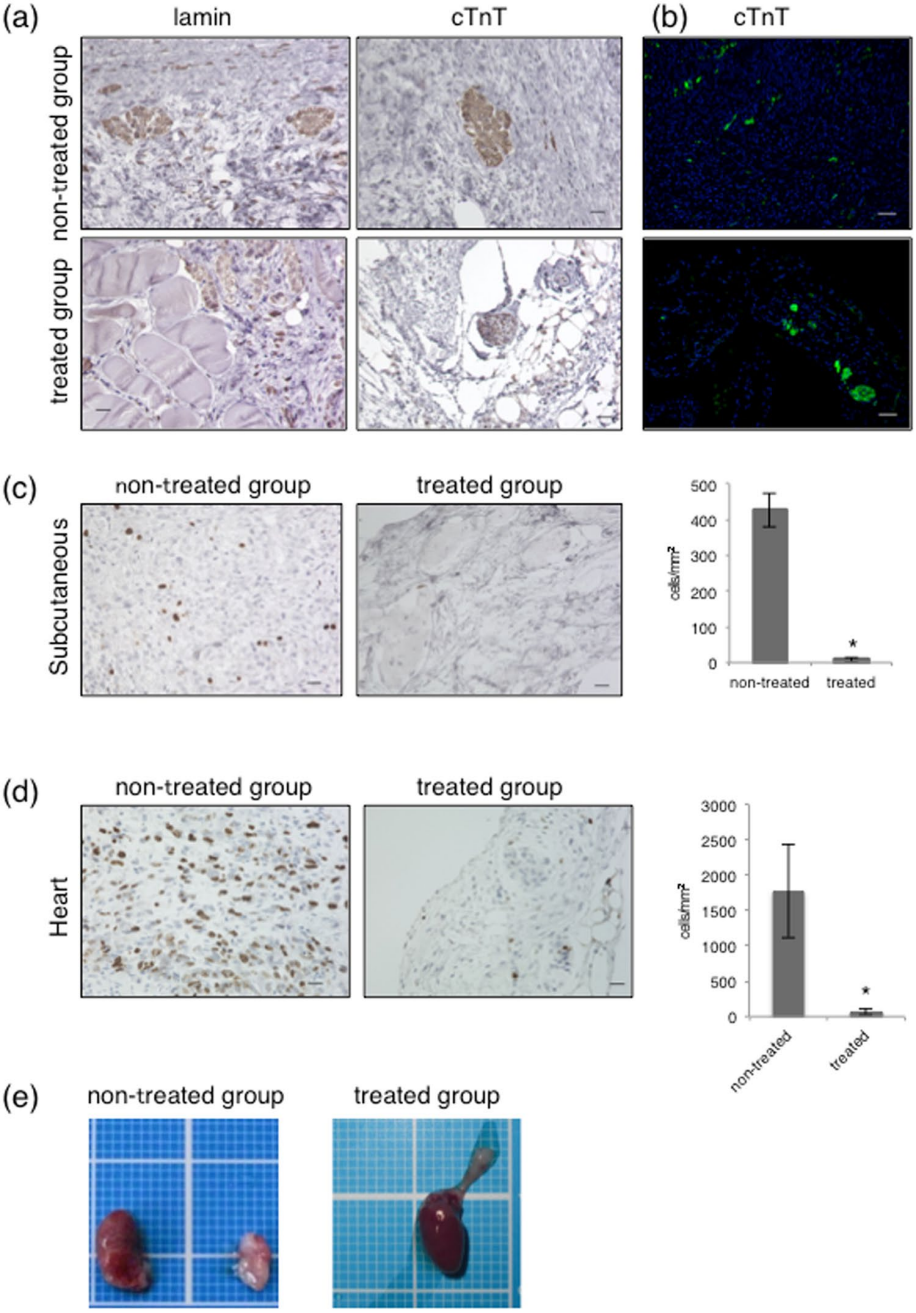
Effect of brentuximab vedotin on teratoma/tumour formation. Teratoma formation in NOG mice 4 months after transplantation of hiPSC-derived CMs with or without brentuximab vedotin treatment. (**a**) Representative images of subcutaneous tissue stained with antibodies against human-specific lamin and cardiac troponin T. Scale bars, 20 μm. Positive cells are seen as dark brown. (**b**) Representative images of cTnT-positive cells. Scale bars, 50 μm. Positive cells are seen as green. (**c**) Representative images of subcutaneous tissue stained with antibody against Ki-67. Scale bars, 20 μm. Positive cells are seen as dark brown. The number of Ki-67–positive cells in the subcutaneous tissue was calculated. *p < 0.01 vs. non-treated group (**d**) Representative images of heart tissue stained with antibody against Ki-67. Scale bars, 20 μm. Positive cells are seen as dark brown. The number of Ki-67 positive cells in the heart was calculated. *p < 0.01 vs. non-treated group (**e**) Representative images of tumour formation in the heart of NOG mice. hiPSC-derived CM sheets with or without brentuximab vedotin treatment were used.

Finally, we subcutaneously injected hiPSC-derived CMs into NOG mice to examine whether brentuximab vedotin can prevent teratoma/tumour formation *in vivo*. After 4 months, no malignant tumour masses developed (Table 1). Human cells surviving in mouse subcutaneous tissues were identified by immunohistochemical analysis. Human-specific lamin antigen-positive cells and cardiac troponin T (cTnT)-positive cells were found in the subcutaneous tissues when hiPSC-derived CMs both with and without brentuximab vedotin treatment were transplanted (Fig. 7a, b). In contrast, a significantly increased number of Ki-67–positive cells was found in the subcutaneous tissues only in the hiPSC-derived CMs without brentuximab vedotin treatment, when compared with hiPSC-derived CMs treated with brentuximab vedotin (424 ± 46.4 cells/mm^2^ and 6.7 ± 5.8 cells/mm^2^, respectively, *p* < 0.005) (Fig. 7c). Similar results were obtained when hiPSC-derived CM sheets with or without brentuximab vedotin treatment were transplanted into the heart. The number of Ki-67–positive cells was significantly reduced in the brentuximab vedotin-treated cell-transplanted group (from 1,778.5 ± 660.7 cells/mm^2^ to 60.7 ± 36.3 cells/mm^2^, *p* < 0.005) (Fig. 7d,e) (Table 1).

**Table 1:** Effect of brentuximab vedotin on malignant tumour formation.

	Subcutaneous	Heart
Non-treated group	9/15 mice (60%)	5/6 mice (83.3%)
Treated group	0/16 mice (0%)	0/8 mice (0%)

Summary of tumour formation with or without brentuximab vedotin in subcutaneous or heart tissue.

## Discussion

In this study, we investigated the effects of brentuximab vedotin on hiPSCs and differentiated cells to confirm its safety in hiPSC therapy. We observed that CD30 was highly expressed on hiPSCs, without detection on differentiated cells such as NHDFs, HCMs, or hiPSC-derived CMs, which strongly suggested that the CD30 antibody-cytotoxic drug conjugate brentuximab vedotin has the potential to remove residual undifferentiated hiPSCs by the mechanism of cell cycle arrest in the G2/M phase. Treatment with brentuximab vedotin induced apoptosis in hiPSCs and significantly reduced the amount of residual undifferentiated cells in hiPSC-derived CMs *in vitro*, leading to prevention of teratoma/tumour formation in NOG mice. The major advantages of this method are 1) it uses a drug which has been approved for clinical application by the FDA, and 2) the drug can be used for not only hiPSC-derived CMs but also numerous other cell lineages derived from hiPSCs.

Cardiomyogenesis using hiPSC-derived CMs is a powerful tool for treatment for heart failure; however, controlling tumourigenicity is crucial for its clinical application. In hiPSC-derived CM-based cell therapy, billions of cells are required for adequate improvement of cardiac performance^3,20^, emphasizing the necessity of developing a new method that promises safety and efficacy in hiPSC-based therapy. Although various approaches such as cell sorting strategies, chemical inhibitors, and introduction of suicide genes have been reported to remove tumourigenic cells, all of them have some limitations in terms of poor efficacy, inconvenience, or high cost. For example, the drawbacks of cell sorting strategies include poor cell viability with low efficacy of separation, which is a great concern in cell therapy that requires a large number of hiPSCs. Regarding chemical inhibitors, there are some concerns regarding selectivity and side effects which may affect the viability of differentiated cells and induce adverse events. The introduction of suicide genes may disrupt endogenous genes, leading to tumourigenic mutations^21^.

Although many new developments have been proposed to solve the problem of tumourigenicity in hiPSCs, the issue remains the approval for clinical use. Brentuximab vedotin is an anti-CD30 chimeric antibody conjugated to the antimicrotubule agent monomethylauristatin E (MMAE). MMAE blocks tubulin polymerization, thus inducing cell death^19,22,23^. Brentuximab vedotin, which is targeted to CD30-positive cells, induced cell cycle arrest in the G2/M phase followed by apoptosis, leading to cytotoxic effects in immature hiPSCs. A previous report indicated that brentuximab vedotin had a strong bystander effect. After binding to CD30-positive cells, brentuximab vedotin is internalized into lysosomes, and then the dipeptide linkers are hydrolyzed by lysosomal enzymes, and free MMAE is subsequently released into the cytoplasm and the extracellular space. CD30-negative cells are as sensitive to free MMAE as CD30-positive cells. A CD30-independent cytotoxic effect on CD30-negative cells is caused by release of free MMAE from neighbouring CD30-positive cells in culture^24^.

In this study, we confirmed that CD30 was highly detected on hiPSCs and was almost undetected on differentiated cells such as NHDFs, HCMs, or hiPSC-derived CMs, in agreement with previous reports^15,25,26^. In addition, CD30 has been reported to be expressed on limited cells in healthy adults^27^. Furthermore, the expression level of CD30 on hiPSCs rapidly diminished with ongoing differentiation into cardiomyocytes. This result corresponds to a previous report describing that the percentage of SSEA4- and TRA-1-81–positive cells was over 50% and that of CD30-positive cells was only 20% or less in partially reprogrammed cell lines^25^. These data suggest that CD30 is a powerful marker of pluripotent cells.

In this study, we also showed that the proportion of hiPSCs with positive expression of CD30 was approximately 90%; therefore, brentuximab vedotin can theoretically remove 90% of hiPSCs. Even though 10% of hiPSCs, i.e., the CD30-negative cells, remained, almost all hiPSCs underwent cell death as shown in Fig. 2 and Fig. 3. We consider that the bystander effect of brentuximab vedotin is likely responsible for these results. However, our study did not fully elucidate whether a bystander effect actually affects the elimination of hiPSCs in this study. Nevertheless, the administration of brentuximab vedotin resulted in complete control of tumourigenicity *in vivo*, suggesting that brentuximab vedotin has some effect on suppressing tumourigenicity in hiPSCs, probably by bystander effects. Alternatively, there is the possibility that CD30-negative hiPSCs have no tumourigenicity. The answer to this question may require further studies.

In conclusion, treatment with brentuximab vedotin induced cell death in CD30-positive hiPSCs and reduced tumourigenicity of hiPSC cardiac derivatives *in vivo*, suggesting that this strategy may increase the safety of clinical applications of hiPSC-based cell therapy for heart disease.

## Methods

### Animals

NOG (NOD/Shi-scid/IL-2Rγnull) mice were obtained from the CIEA (Central Institute for Experimental Animals, Kawasaki, Japan). The animal care procedures complied with the “Guide for the Care and Use of Laboratory Animals” (NIH publication no. 85–23, revised 1996). All animal experiments were performed under an experimental protocol approved by the Ethics Review Committee for Animal Experimentation of the Osaka University Graduate School of Medicine.

### Cell culture

The human iPSC lines 201B7^1^, 253G1^28^, Ff-I01, and Ff-I14^29^ were provided by the Center for iPS Cell Research and Application, Kyoto University. 201B7 and 253G1 hiPSCs were cultured on Matrigel-coated dishes with mTeSR1 (Stem Cell Technologies, Vancouver, Canada) under standard cell culture conditions (37 °C, 5% CO_2_). Ff-I01 and Ff-I14 cells were cultured on iMatrix-511 (Nippi, Inc., Tokyo, Japan)-coated dishes with StemFit (Ajinomoto Co., Inc., Japan)^29^. Normal human dermal fibroblasts (NHDFs) (Lonza, Walkersville, MD) were cultured under standard cell culture conditions in Dulbecco’s modified Eagle medium (DMEM) (Nacalai Tesque, Kyoto, Japan) containing 10% foetal bovine serum (FBS). Human cardiac myocytes (ScienCell, Carlsbad, CA) were cultured under standard cell culture conditions in cardiac myocyte medium with cardiac myocyte growth supplement and penicillin/streptomycin solution containing 5% FBS (ScienCell).

### Cardiac differentiation in a bioreactor

Cardiac differentiation was performed in a bioreactor as previously described^30^. In brief, one day after starting cultures in the bioreactor system, hiPSCs were cultured in StemPro34 medium (GIBCO, Grand Island, NY) containing 50 μg/ml ascorbic acid (Wako, Miyazaki, Japan), 2 mM L-glutamine (GIBCO), and 400 μM L-thioglycerol (Sigma-Aldrich) with 0.5 ng/ml BMP4 (R&D systems) (Day 0). The following growth factors and small molecules were used at the corresponding days: Day 1, 10 ng/ml BMP4, 5 ng/ml bFGF (ReproCELL, Tokyo, Japan), 3 ng/ml activin A (R&D systems); Day 4, 4 μM IWR-1 (Sigma- Aldrich); Day 6, 8, 10, 12, and 14, 10 ng/ml bFGF and 5 ng/ml VEGF (R&D systems). At Days 4, 6, 8, 10, 12, and 14, the culture medium was replaced with fresh medium. At Day 16, cells were collected.

### Flow cytometric analysis

The cells were resuspended in FACS buffer [PBS with 0.5% bovine serum albumin (Sigma), 0.4% Block Ace (DS Pharma Biomedical, Osaka, Japan), and 10 mM EDTA]. For screening of cell surface markers, cells were subjected to BD Lyoplate screening panels (BD Bioscience, San Jose, CA). For cell surface antigens, 5 × 10^5^ cells were washed with PBS and incubated in FACS buffer containing 10% human serum for 20 min on ice. PE-conjugated anti-CD30 antibody (BD) and FITC-conjugated anti-TRA-1-60 antibody (BD) were added for 30 min, after which the cells were washed and resuspended in FACS buffer. The samples were then assayed by flow cytometry (FACSCanto II, BD Biosciences) and the results were analysed using BD FACSDiva software (BD Biosciences). More than 10,000 events were analysed and compared with isotype controls.

### Cell viability assay

Cells were added to a 12-well plate (1 × 10^5^ cells/well) with 900 μl of medium and Y-27632 (final concentration 10 μM, Wako, Osaka, Japan) and incubated at 37 °C, 5% CO_2_. The next day, the medium was replaced with fresh medium without Y-27632. After 24 h, fresh medium with or without brentuximab vedotin (ADCETRIS^TM^, Takeda, Osaka, Japan) was added and incubated for 24, 48, 72, and 96 h. Then, supernatants were discarded and fresh medium with CCK-8 (Cell Counting Kit-8) solution (Dojindo, Kumamoto, Japan) was added and incubated for 2 h under standard cell culture conditions (37 °C, 5% CO_2_). The absorbance at 450 nm was then recorded using a Power Scan H1 fluorescence plate reader (DS Pharma Biomedical). Wells with the medium alone (no cells) served as blank controls. Viability was calculated from the absorbance of treated cells relative to untreated cells.

### Cell cycle assay

For cell cycle analysis, cells were incubated for 10-12 h with or without brentuximab vedotin at the indicated concentrations. Subsequently, the cells were collected, washed with PBS, and fixed with ice-cold 70% ethanol at 4 °C for 30 min. The cells were then washed with PBS and incubated with propidium iodide (PI) (BD) (50 μg/ml) and RNaseA (Invitrogen, Carlsbad, CA) (50 μg/ml). Cell cycle analysis was performed on a FACSCanto II system (Becton Dickinson, San Jose, USA) using BD FACSDiva software.

### Apoptosis assay

Apoptosis assays were performed using annexin V-FITC and 7-AAD. Cells were treated with different concentrations of brentuximab vedotin for 48 h, collected, washed with 1 × PBS, and stained with annexin V-FITC and 7-AAD. Analysis was performed on a FACSCanto II system using BD FACSDiva software.

### Quantitative real-time polymerase chain reaction

Total RNA was extracted using a PureLink RNA Mini Kit (Ambion, Carlsbad, CA) and reverse-transcribed using SuperScript III Reverse Transcriptase (Invitrogen) with random primers (Invitrogen). The quantitative real-time polymerase chain reaction was performed using the ViiA7 Real-Time PCR System (Applied Biosystems, Carlsbad, CA). Each sample was analysed in triplicate for each gene study. Data were normalized to the expression levels of glyceraldehyde-3-phosphate dehydrogenase (GAPDH). Primer sequences for Lin28 are: 5′-CACGGTGCGGGCATCTG-3′ (forward) and 5′-CCTTCCATGTGCAGCTTACTC-3′ (reverse)^31^, and primer sequences for GAPDH are 5′-CGATGCTGGCGCTGAGTAC-3′ (forward) and 5′-CCACCACTGACACGTTGGC-3′ (reverse).

### LDH release assay

LDH activity was determined using a LDH Cytotoxicity Detection Kit (Takara, Ohtsu, Japan). In brief, hiPSC-derived CMs were cultured in appropriate plates in DMEM supplemented with 10% FBS at 37 °C, 5% CO_2_, overnight. Then, supernatants were discarded, and various concentrations of brentuximab vedotin were added and incubated for 72 or 96 h. After incubation, the amount of LDH in the supernatant was measured. Non-treated cells were used as a control.

### Cell motion analysis

Cells were seeded with Y-27632 and incubated at 37 °C under 5% CO_2_. The next day, the medium was replaced with fresh medium without Y-27632. After 24 h, fresh medium with or without brentuximab vedotin was added and incubated for 24, 48, 72, and 96 h. Cells were monitored at a frame rate of 150 fps for 10 seconds at 37 °C with a high-speed 4-camera-based motion analysis system (SI8000 View; Sony, Tokyo, Japan). Data were analysed using a SI8000C analyser (Sony).

### Cell sheet preparation

Prior to seeding of cells, the surface of temperature-responsive dishes (UpCell; CellSeed, Japan) was coated with FBS for 2 h. After cardiac differentiation, cells were dissociated with 0.05% trypsin/EDTA, cell aggregates were removed using a strainer, and single cells were plated onto the UpCell at 5.5 × 10^5^ cells/cm^2^ in DMEM supplemented with 10% FBS and cultured at 37 °C under 5% CO_2_. After 72 h in culture, hiPSC-CM sheets were harvested by incubation at room temperature and washed gently with HBSS (+).

### Teratoma formation assay

hiPSC-derived CMs were transplanted into 7- or 8-week-old male NOG mice. Prior to seeding of cells, dishes were coated with 0.1% gelatin at 37 °C for at least 60 min. For subcutaneous transplantation, 9.5 × 10^6^ hiPS-CMs were seeded onto 6-cm dishes in DMEM supplemented with 10% FBS and 1% penicillin-streptomycin, and then brentuximab vedotin (10 μg/ml) was added. After 72 h of culture, hiPSC-CMs were harvested and then injected into the subcutaneous tissue of NOG mice. For transplantation into the heart, 5 × 10^6^ hiPS-CMs were seeded onto 3.5-cm dishes in DMEM supplemented with 10% FBS and 1% penicillin-streptomycin, and then brentuximab vedotin (10 μg/ml) was added. After 72 h in culture, hiPSC-CM sheets were harvested by incubation at room temperature and washed gently with HBSS (+), and then placed onto the heart of NOG mice. Four months after transplantation, mice were sacrificed and tissues were harvested, fixed with 10% formalin overnight, and embedded in paraffin using a Microm STP 120 Spin Tissue Processor (STP120-3, Thermo Fisher Scientific Inc., Massachusetts). The tissues were sectioned at 0.5 µm using a Microm HM 430 system (MIC 990010, Thermo Fisher Scientific Inc.) and stained with haematoxylin and eosin. Immunostaining was performed using anti-Ki-67 antibody (mouse monoclonal antibody, clone MIB-1) (Dako, Denmark) and anti-lamin antibody (rabbit monoclonal antibody, clone EPR4100) (Abcam, Cambridge, UK). Briefly, after inactivation of endogenous peroxidase, the slides were incubated overnight at 4 °C with primary antibodies. The slides were then incubated with secondary antibody for 30 min at 37 °C, after which they were incubated with labelled streptavidin biotinylated antibody for 30 min at 37 °C, and then visualised following incubation with 3,3′-diaminobenzidine. Next, the slides were counterstained with Mayer’s haematoxylin for 5 min at room temperature. The slides were visualized under a microscope.

### Statistical analysis

Data are expressed as the mean ± SEM values. The data distributions were checked for normality. Comparisons between two groups were performed using Student’s t-test. All P values were two-sided, and p < 0.05 was considered to indicate statistical significance.
